# Arsenic Methyltransferase and Apolipoprotein E Polymorphism in Pregnant Women Exposed to Inorganic Arsenic in Drinking Water in Western Romania

**DOI:** 10.3390/ijms25063349

**Published:** 2024-03-15

**Authors:** Laura Ancuta Pop, Ioana Berindan-Neagoe, Michael S. Bloom, Iulia Adina Neamtiu, Cecilia Bica, Eugen S. Gurzau

**Affiliations:** 1 Research Center for Functional Genomics, Biomedicine and Translational Medicine, Iuliu Hatieganu University of Medicine and Pharmacy, 8 Victor Babes Street, 400012 Cluj-Napoca, Romania; laura.ancuta.pop@gmail.com (L.A.P.); egurzau@ehc.ro (E.S.G.); 2 Department of Global and Community Health, George Mason University, 4400 University Dr, Fairfax, VA 22030, USA; mbloom22@gmu.edu; 3 Health Department, Environmental Health Center Part of ALS, 58 Busuiocului Street, 400240 Cluj-Napoca, Romania; 4 Faculty of Environmental Science and Engineering, Babes-Bolyai University, 30 Fantanele Street, 400294 Cluj-Napoca, Romania

**Keywords:** inorganic arsenic, environmental exposure, genotyping, pregnancy, single-nucleotide polymorphism

## Abstract

Previous studies have shown that inorganic arsenic (iAs) exposure may be associated with genotoxic and cytotoxic effects. The aim of this study was to evaluate the relationship between several polymorphisms in *AS3MT* and *APOE* genes and urinary As and the relationship between these polymorphisms and pregnancy loss. We determined urinary As concentrations and performed genotyping analysis in 50 cases of spontaneous pregnancy loss and 50 controls, matched to cases on gestational age. The most frequently identified *AS3MT* polymorphisms in both cases and controls were in rs10748835 (80% cases and 68% controls), rs3740400 (78% cases and 64% controls), rs7085104 (74% cases and 48% controls), and rs1046778 (62% cases and 54% controls). We identified 30 different haplotypes in *AS3MT* SNPs, with four predominant haplotypes (>8%). Cases with Haplotype 1 had four-fold higher urinary DMA and two-fold higher MMA concentration than those without this haplotype, the MMA levels were lower in cases and controls with Haplotype 4 compared to Haplotype 1, and the DMA levels were significantly lower in cases with Haplotype 4 compared to Haplotype 3. Cases with Haplotype 1 had higher levels of all analyzed biomarkers, suggesting that Haplotype 1 may be associated with greater exposure to iAs and tobacco smoke. Our results suggest the importance of the *AS3MT* gene in iAs metabolism among pregnant women with low-level drinking water iAs exposure.

## 1. Introduction

Pregnancy loss affects approximately 15% of pregnant women [1], and it has been related to several risk factors, including genetic factors, medical history, environmental exposure, and others [2,3]. Studies conducted worldwide have shown that greater exposure to inorganic arsenic (iAs) was associated with pregnancy loss [4]. Arsenic (As) is a toxic metalloid found in soil, sediments, or ground water, which can contaminate drinking water and/or food. It is estimated that over 200 million people worldwide are exposed to As concentrations in drinking water exceeding the World Health Organization (WHO) and US Environmental Protection Agency (US EPA) maximum contaminant level (MCL) of 10 μg/L [5,6]. In the human body, iAs is metabolized by methylation. The enzyme As methyltransferase (AS3MT) is involved in iAs metabolism and transforms iAs into its metabolites, monomethylarsonic acid (MMA) and dimethylarsinic acid (DMA) [7,8]. Previous studies have shown that MMA^III^ and DMA^III^ may be associated with genotoxic and cytotoxic effects [8,9] and that their relative urinary concentrations may be related to the efficiency of AS3MT activity. In addition, iAs can cross the placenta, affecting fetal development, and prenatal maternal exposure to high concentrations of As in drinking water was associated with higher risks of adverse pregnancy outcomes, including miscarriage and fetal death [10,11].

Inorganic As metabolism can be influenced by genetic differences, which may predispose individuals to adverse health outcomes related to iAs exposure [12]. Previous studies have shown that polymorphisms in the *AS3MT* gene may be associated with reduced gene expression and enzymatic activity, which were correlated with urinary MMA and DMA levels [13]. Other studies reported that single-nucleotide polymorphisms (SNPs) in the *AS3MT* gene were related to iAs exposure in drinking water and were associated with lower percentages of MMA and higher percentages of DMA in urine [14,15,16]. The rs3740393 and rs11191439 polymorphisms were associated with iAs methylation, the former affecting the second step of methylation and the latter affecting the first step of methylation [17]. Skin cancer patients with rs3740400C, rs3740393C, rs11191439T, and rs1046778C and exposed to iAs via contaminated drinking water had lower percentages of urinary MMA and iAs and a higher percentage of urinary DMA, while those with rs3740400C, rs3740393G, rs11191439C, and rs1046778T and exposed to iAs via contaminated drinking water had lower urinary DMA and higher urinary MMA percentages [18]. A study of maternal *AS3MT* SNPs and fetal development showed that rs7085104, rs3740400, rs3740393, rs3740390, and rs1046778 were associated with urinary iAs concentration and that rs340393 was associated with adverse birth outcomes [19].

The apolipoprotein E (*APOE*) gene, which plays a role in lipid metabolism and regulates steroid hormone functions [20,21], has been associated with recurrent pregnancy loss. The *APOE* gene has three isoforms encoded by alleles ε2, ε3, and ε4. Studies have shown that carriers of the *APOE* ε4 isoform are more susceptible to metal toxicity than those without the *APOE* ε4 isoform, which may affect embryo development [22,23]. The *APOE* SNPs rs7412 and rs429358 were identified as potential risk factors for pregnancy loss in Bosnian women [24] and also were associated with higher As levels in urine and cord blood in a Croatian population [25].

Although previous studies have been conducted, the associations between *AS3MT*, *APOE* SNPs, and iAs exposure remain unclear among women with pregnancy loss. The aim of this study was to evaluate the relationship between several polymorphisms in the *AS3MT* and *APOE* genes and urinary iAs concentrations as well as the differences between these polymorphisms in women with and without spontaneous pregnancy loss.

## 2. Results

Table 1 presents the distribution of lifestyle factors and urinary biomarkers of As and tobacco smoke exposure among study participants, by case status. Median urinary DMA and MMA levels were higher in cases than controls.

**Figure 1:**
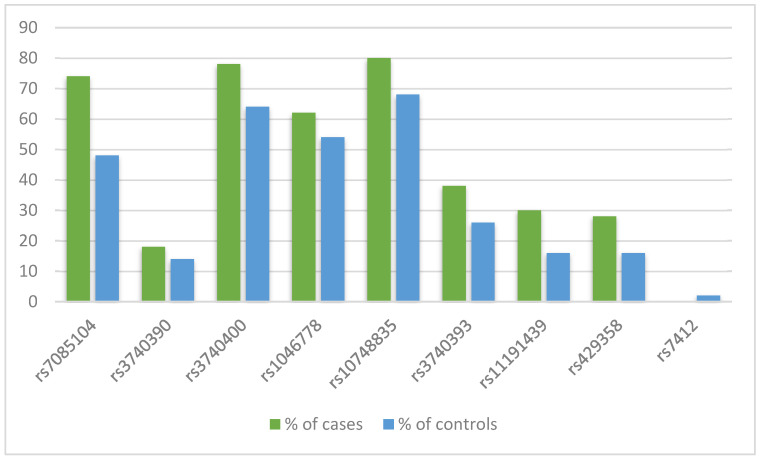
Distribution of identified polymorphisms by case status.

The mutation percentages for the nine analyzed polymorphisms are presented in Figure 1. Five cases and 12 controls had no alteration in the tested polymorphisms. Overall, the analysis showed more cases with the tested polymorphisms than controls, and the rs7412 was observed only in one control as a heterozygous alteration (Figure 1). Also, our results showed that the most frequently identified alterations in both cases and controls were in rs10748835 (80% cases and 68% controls), rs3740400 (78% cases and 64% controls), rs7085104 (74% cases and 48% controls), and rs1046778 (62% cases and 54% controls) (Figure 1).

The analysis of specific genotype of the identified polymorphisms indicated that all subjects with mutations in rs7085104, rs3740390, and rs1046778 had heterozygous genotypes, while for the other polymorphisms, they also had homozygous genotypes (Table 2).

**Figure 2:**
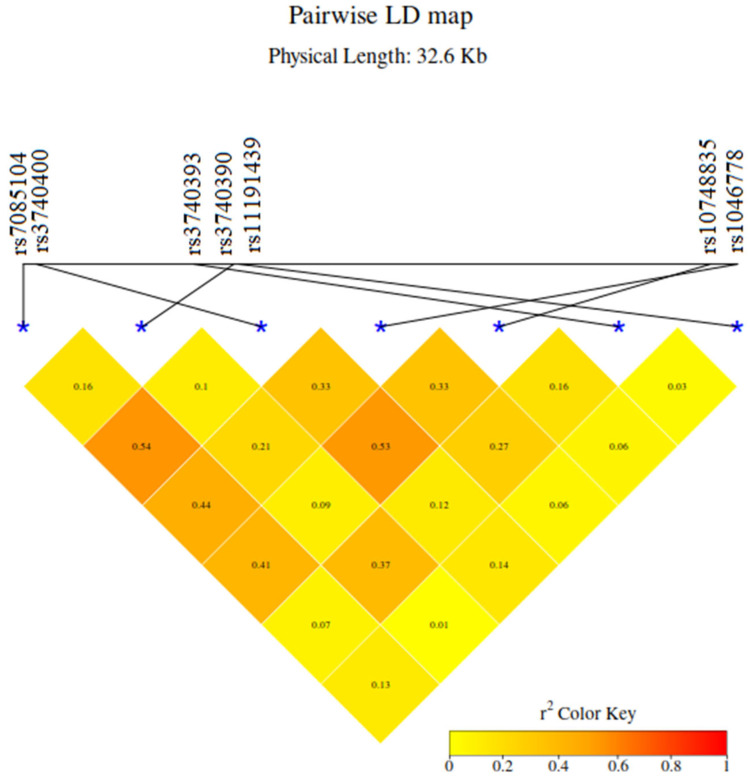
Linkage disequilibrium analysis for *AS3MT* single-nucleotide polymorphisms in cases and controls. R^2^—squared correlation between allelic values at two loci; R^2^ = 0 (yellow) indicates weak correlation; R^2^ = 1 (red) indicates strong correlation. Abbreviation: LD = linkage disequilibrium. “*” indicates where each SNP is represented on the graph.

All SNPs were in the Hardy–Weinberg equilibrium. We also analyzed the linkage disequilibrium (LD values (R^2^)) for the tested SNPs. As shown in Figure 2, we found that rs7085104 was in a moderate LD with rs3740400, rs1046778, and rs10748835 (R^2^ between 0.54 and 0.41); rs3740390 was in a moderate LD with rs3740393 (R^2^ = 0.37); rs3740400 was in a moderate LD with rs10748835 and rs1046778 (R^2^ between 0.53 and 0.33); and rs1046778 was in a moderate LD with rs10748835 and rs3740393 (R^2^ between 0.33 and 0.27) in our study participants. These results show that each of the six SNPs can act as a proxy for the other, and that they generally are inherited together. Rs11191439 was not in an LD with the other tested SNPs.

Table 3 shows the identified haplotypes and the numbers and percentages of cases and controls that had a specific haplotype. We identified 30 different haplotypes for the *AS3MT*-tested SNPs, with 4 predominant haplotypes having a frequency higher than 8%. There were 7 haplotypes specific to controls and 15 haplotypes specific to cases. Twelve haplotypes were observed only in one case and seven only in one control.

For cases, there were four haplotypes with a frequency higher than 12%, while for controls three haplotypes appear to be more frequent (i.e., >12%). In addition, seven cases and ten controls had haplotypes composed of wildtype alleles (H002), while all other haplotypes contained at least one mutant allele.

**Figure 3:**
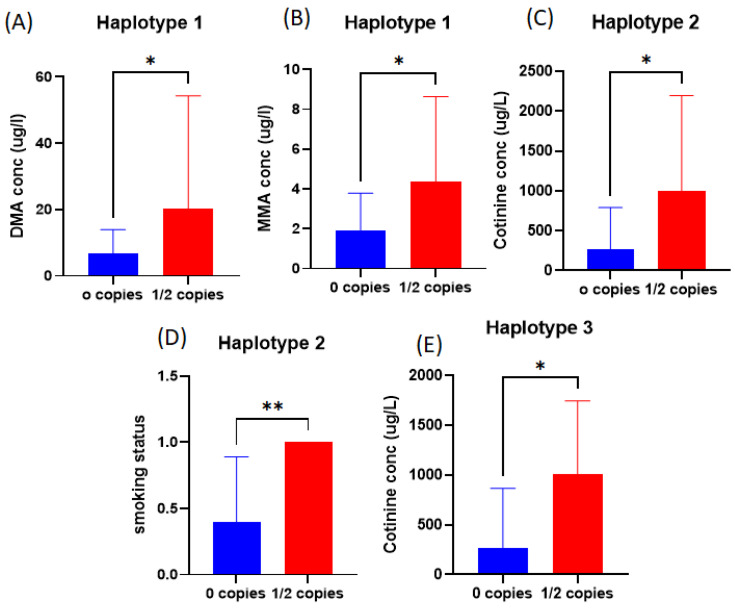
Comparison analysis of the biomarkers of exposure to iAs and tobacco smoke in cases with different *AS3MT* haplotypes: (**A**) Bar graph describing the difference in DMA concentrations between cases with and without Haplotype 1; (**B**) Bar graph describing the difference in MMA concentrations between cases with and without Haplotype 1; (**C**) Bar graph describing the difference in cotinine concentrations between cases with and without Haplotype 2; (**D**) Bar graph describing the difference in smoking status of cases with and without Haplotype 2; (**E**) Bar graph describing the difference in cotinine concentration between cases with and without Haplotype 3 The analysis was performed using the *t*-test to compare between cases with 1/2 copies of the specific haplotype versus cases with no copies of the specific haplotype (*p*-value < 0.05 indicates statistically significant results) * *p* value < 0.05, ** *p* value = 0.0095. Abbreviations: MMA, monomethylarsonic acid; DMA, dimethylarsinic acid.

We further analyzed the relationships between different *AS3MT* haplotypes, urinary iAs, and self-reported exposure to tobacco smoke and found that cases with Haplotype 1 had higher urinary DMA and MMA concentrations than cases without this haplotype (Figure 3A,B). Also, our analysis showed a higher urinary concentration of cotinine in cases with Haplotype 2 or 3 than in cases without these haplotypes (Figure 3C,E). Furthermore, cases with Haplotype 2 were predominantly smokers (self-reported), as shown in Figure 3D.

**Figure 4:**
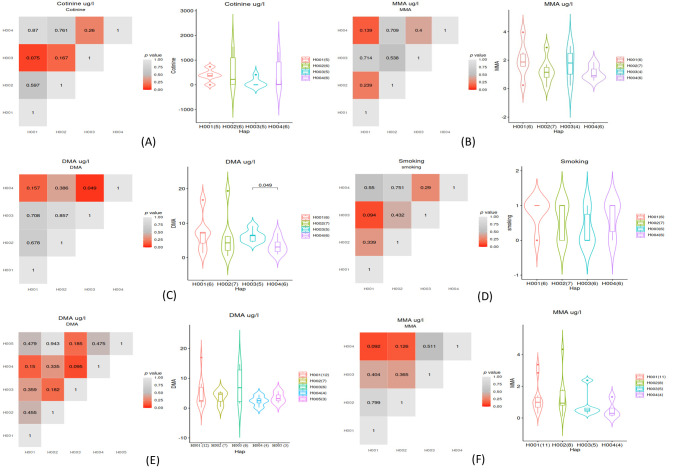
Comparison analysis of the biomarkers of exposure to iAs and tobacco smoke, by case status and the identified *AS3MT* haplotypes: (**A**) urinary cotinine for cases; (**B**) MMA for cases; (**C**) DMA for cases; (**D**) smoking status for cases; (**E**) DMA for controls; and (**F**) MMA for controls. The analysis was performed using the *t*-test to compare between the identified haplotypes. *p* value < 0.05 indicates a statistically significant result. Abbreviations: MMA, monomethylarsonic acid; DMA, dimethylarsinic acid.

We also investigated the relationships between the identified haplotypes and urinary iAs and tobacco smoke exposure as shown in Figure 4. The analysis showed that cases with Haplotype 3 had lower urinary levels of cotinine than cases with Haplotype 1, but the difference was not statistically significant (*p* = 0.075). Urinary MMA levels were lower in cases and controls with Haplotype 4 compared to those with Haplotype 1, albeit not statistically significant (*p* = 0.139 and *p* = 0.092, respectively), suggesting that cases with Haplotype 1 might have a higher degree of methylation that those with Haplotype 4. Urinary DMA levels were significantly lower in cases with Haplotype 4 compared to cases with Haplotype 3 (*p* = 0.049), while in controls this difference was not statistically significant (Figure 4).

## 3. Discussion

We found that the most frequently identified *AS3MT* alterations in cases of spontaneous pregnancy loss and controls with ongoing pregnancies were in rs10748835, rs3740400, rs7085104, and rs1046778, and there was a higher number of cases with the tested polymorphisms than controls. Also, rs7412 was identified only in one control as a heterozygous alteration. The analysis of haplotypes identified four predominant haplotypes with a frequency >8% for the *AS3MT* tested SNPs. Seven cases and ten controls had haplotypes composed of wildtype alleles, while all other haplotypes contained at least one mutant allele. We found that cases with Haplotype 1 had four-fold higher urinary DMA and two-fold higher MMA concentration than those without Haplotype 1, MMA levels were lower in cases and controls with Haplotype 4 compared to Haplotype 1, and DMA levels were significantly lower in cases with Haplotype 4 than Haplotype 3, although the association was not statistically significant in controls. Cases with Haplotype 1 had higher urinary levels of all analyzed biomarkers than cases without Haplotype 1. This study conducted in a population with low levels of drinking water iAs contamination showed that polymorphisms in *AS3MT* were associated with urinary iAs metabolites, suggesting an association with iAs metabolism. We previously measured iAs in 124 public and private drinking water sources, including wells and taps, used by women in our study. Levels of iAs in water sources were low overall (between <0.5–175 µg/L; median = 3.0 µg/L) although higher in wells (between <0.5–1.75 µg/L; median = 3.1 µg/L) than in community taps (between <0.5–36.4 µg/L; median = 2.7 µg/L) [26]. However, our study results were consistent with the results from other studies on population groups exposed to medium and high levels of iAs in drinking water [16,27,28,29,30,31,32]. Engstrom et al. reported that different haplotypes of tested *AS3MT* SNPs had different effects on the methylation and expression profile of *AS3MT* and other genes in an Argentinian study population with high levels of drinking water iAs contamination [33]. Collectively, the results suggest that *AS3MT* haplotypes are relevant to iAs metabolism across a wide range of plausible human drinking water exposure.

We found that the minor allele of the selected SNPs was more frequent in cases compared to controls, which may play an important role in evaluating the effect of co-exposure to iAs and tobacco smoke on pregnancy loss. However, given our limited sample size, a larger study will be necessary to validate the result. Also, we identified more haplotypes in cases compared to controls. Similar to our results, other studies reported that SNPs in the *AS3MT* gene were associated with altered iAs metabolism efficiency [16,34]. A previous study conducted in Arad and Bihor counties, Romania, reported that four SNPs (rs3740400, rs3740393, rs11191439, and rs1046778) in *AS3MT* generated seven different haplotypes with a frequency between 1 and 57%, with haplotype AGTT being the most frequent [18]. Another study conducted in a population group from Central Europe, reported that rs11191439 had an influence on iAs metabolism [27].

Our analysis showed that Haplotype 1 in cases was associated with higher urinary DMA and MMA concentrations than in other haplotypes. Also, we showed that the minor alleles of four of the tested SNPs were associated with higher urinary concentration of MMA than in other haplotypes, which suggests a higher risk of an elevated urinary MMA phenotype, as was also reported by a previous study [19]. High urinary MMA concentrations have been associated with DNA damage and adverse pregnancy outcomes in prior studies [33,35]. The major alleles of the tested SNPs were associated with higher urinary cotinine and self-reported smoking, a risk factor for pregnancy loss [36].

The *APOE* gene can influence oxidative stress, metalloid kinetics, and neurodevelopment [22,34,37,38] and several studies indicated that the presence of *APOE* variance was associated with pregnancy loss [21,39,40]. In our study, we identified *APOE* rs429358 in 14 cases, but we did not find a correlation between this SNP and urinary DMA, MMA, or cotinine concentrations. This result was consistent with previous results showing no correlation between pregnancy loss and *APOE* SNPs [24].

Our study has several strengths. To our knowledge, this is one of few studies that reports an association between different *AS3MT* polymorphisms and urinary biomarkers of exposure to iAs via contaminated drinking water (MMA and DMA levels) and tobacco smoke (cotinine levels). Also, this is one of the first studies to analyze the association between pregnancy loss and *AS3MT* and *APOE* polymorphisms.

However, there are important limitations to our study. The small sample size in our study and the small *n* for haplotypes had limited statistical power to detect differences. Another limitation was related to the quantity and quality of the DNA obtained after the extraction, leading to a lower amplification of some samples. Also, we determined urinary iAs metabolites from a spot urine sample collected at the time of loss for cases or during ongoing pregnancies for controls. This may have misclassified urinary iAs measurements for some women, as the proportion of DMA to MMA tends to rise later in pregnancy [10]. Still, this measurement error was probably random in our analysis, with a bias towards the null hypothesis.

## 4. Material and Methods

### 4.1. Study Participants and Biological Specimen Collection

A detailed description of study participant enrollment was provided in a previous publication [41]. Briefly, study participants were recruited from women receiving prenatal care services at the Obstetrics and Gynecology Department of the Emergency County Hospital (Bega Hospital) in the city of Timisoara (Timis County, Romania). The study included 50 women with clinically recognized spontaneous pregnancy losses prior to 20 weeks’ completed gestation as case participants and 50 women with ongoing singleton pregnancies of similar duration to each case participant (±1 week) as control participants. Written informed consent was provided by each participant before enrollment in the study. The research protocol was approved by the Institutional Review Boards of the County Emergency Hospital in Timisoara (IRB #40/09.29.2011) and the University at Albany, State University of New York (IRB #0000590, protocol #11-239). All study participants completed a physician-administered questionnaire collecting information on demographic, socioeconomic, and lifestyle factors and medical, gynecologic, and occupational histories. At the time of enrollment, spot urine samples were collected by study nurses in 50 mL polyethylene containers previously decontaminated with nitric acid. The collected urine samples were frozen within 15 min of collection and stored at −20 °C until analysis. Venous blood specimens were also collected in 2 mL vacutainers and stored at −20 °C until analysis [26].

### 4.2. Urinary as Analysis

Urinary As was determined in *n* = 222 urine specimens using a PlasmaQUANT MS ELITE Inductively Coupled Plasma—Mass Spectrometer (ICP-MS; Analytik Jena, Jena, Germany) coupled with a SYCAM High Performance Liquid Chromatography (HPLC) system (Sykam GmbH, Eresing, Germany). We measured urinary concentrations of trivalent As (As III), pentavalent As (As V), MMA, DMA, and arsenobetaine as µg/L. We described the analytical method for urinary iAs determination in our previous publication [42]. Briefly, we used deionized water, ammonium carbonate (NH_4_)_2_CO_3_, and methanol for the analysis. For validation of the analytic method and determination of As species in urine, we used National Institute of Standards and Technology (NIST)-traceable As III and As V ICP reference materials (RMs) and arsenobetaine, MMA, and DMA As species in frozen human urine -NIST2669/Standard Reference Material (SRM). The samples were diluted 1:10 with a solution of 6 mM (NH_4_)_2_CO_3_, and 10 µL of the prepared solution was injected into the HPLC system using a HPLC SYKAM S5250 autosampler. A five-point calibration curve was plotted for each determination of each As species. The method limit of detection (LOD) was 0.1 µg/L for all As species. Quality assurance/quality control (QA/QC) was maintained by analyzing a control sample of known concentration prepared from a certified RM for each As species and a blank sample every 10 samples. Also, every 10 samples, a replicate or duplicate sample was prepared and analyzed. A sample of certified RM (As species in frozen human urine NIST 2669) was prepared and analyzed for each set of determinations, as well.

### 4.3. Analysis of Cotinine in Urine

We determined cotinine in urine as a biomarker of tobacco smoke exposure [43], which is also a source of exposure to iAs [44]. Cotinine was extracted with dichloromethane, evaporated to dryness, dissolved in toluene, and analyzed on a gas chromatograph Shimadzu GS (Shimadzu, Kyoto, Japan) in SIM mode, coupled to a Shimadzu GC-MS TQ8040 mass spectrometer (Shimadzu, Kyoto, Japan) and a Shimadzu AOC 20i autosampler (Shimadzu, Kyoto, Japan). Further details about the method can be found in our previous publications [42,45].

### 4.4. DNA Isolation and Genotyping Analysis

We performed genotyping analysis in 50 randomly selected case pregnancies and 50 control pregnancies, matched to cases on gestational age. DNA was isolated from 200 µL of whole blood using the QiAmp DNA Blood kit (Qiagen, Hilden, Germany) according to the manufacturer’s instructions. The DNA samples were quantified using Nanodrop. The genotyping analysis was performed on the QIAcuity Digital PCR (Qiagen, Hilden, Germany) system using specific dPCR LNA Mutation Assay (Qiagen, Hilden, Germany) for the following polymorphisms: rs7085104, rs3740390, rs3740400, rs1046778, rs10748835, rs3740393, rs11191439, rs429358, and rs7412. We used 250 ng of DNA for each reaction and added 10 µL of 4XProbe PCR mix, 1.3 µL of 30X-dPCR LNA assay (Qiagen, Hilden, Germany), and 0.5 µL of restriction enzyme (EcoRI). The mixture was placed in the QiAcuity Digital PCR system (Qiagen, Hilden, Germany) using the following program: 1 cycle 2 min 95 °C, 40 cycles 15 s 95 °C, and 30 s 60 °C. When the PCR analysis was completed, the plate was read for FAM and VIC fluorophores, and the results were analyzed using the instrument’s software.

All blood samples were analyzed for nine polymorphisms. Six of the seven SNPs for the *AS3MT* gene are in intronic regions, while rs11191439 is in the gene exon nine. Rs429358 and rs7412 are in exon four of the *APOE* gene.

### 4.5. Statistical Analysis

Statistical analysis was performed using Graph Pad Prism v.10.0.2 for Windows (GraphPad Software, Boston, MA, USA). The relationships between the identified polymorphisms and the urinary iAs biomarkers and urinary cotinine were estimated using Pearson’s correlation coefficients among cases and among controls. Haplotype analysis and the haplotype–phenotype association analysis (i.e., correlations between genetic haplotype and the corresponding phenotypic traits) were performed using the “geneHapR” package in R. Linkage disequilibrium analysis was performed in R using the “GWLD” package (a package for genome wide linkage disequilibrium analysis) and the linkage disequilibrium (LD) was estimated using R^2^ values.

The R version 4.2.2 Statistical Computing Environment (R Foundation for Statistical Computing, Vienna, Austria) was used for all analyses.

## 5. Conclusions

We report a higher number of cases with the tested polymorphisms than controls. We found that cases with Haplotype 1 had higher urinary DMA and MMA concentrations than other haplotypes. In addition, urinary MMA levels were lower in cases and controls with Haplotype 4 compared to Haplotype 1, although not statistically significant, and urinary DMA levels were significantly lower in cases with Haplotype 4 compared to those with Haplotype 3. Also, cases with Haplotype 1 had higher urinary levels of all biomarkers analyzed, suggesting that Haplotype 1 may be associated with greater exposure to iAs and tobacco smoke than other haplotypes.

Although this study did not identify a specific molecular mechanism of the effect of iAs exposure on pregnancy loss, our results suggest the importance of the *AS3MT* gene in iAs metabolism among pregnant women. Larger studies are necessary to elucidate specific molecular mechanisms of that drive the associations between iAs exposure and pregnancy outcomes.

## Figures and Tables

**Table 1:** Distribution of sociodemographic factors, health-related behaviors and biomarkers of As exposure among study participants, by case status (*n* = 100).

Factor	Cases (*n* = 50)				Controls (*n* = 50)			
	*n*	Median (%)	Range	25–75% Tile	*n*	Median (%)	Range	25–75% Tile
Smoked during pregnancy								
Never smoker	28	(56.0)			25	(50.0)		
No	9	(18.0)			10	(20.0)		
Yes	13	(26.0)			15	(30.0)		
Urinary cotinine ^a^ (μg/L)	50	<5 ^b^	<5–4164	<5–407.7	49	<5	<5–2219.1	<5–496.6
MMA (μg/L)	50	1.5	<0.1 ^c^–10.2	0.8–2.9	50	0.9	<0.1–644	0.5–1.5
DMA (μg/L)	50	5.2	0.4–89.6	3.0–7.4	50	3.7	<0.1–74.9	1.8–7.1

^a^ *n* = 1 missing control value; ^b^ limit of detection for urinary cotinine; ^c^ limit of detection for urinary MMA. Abbreviations: As, arsenic; DMA, dimethylarsinic acid; MMA, monomethylarsonic acid.

**Table 2:** Distribution of specific *AS3MT* and *APOE4* genotypes in rs3740400, rs10748835, rs3740393, rs11191439, and rs429358 by case status.

Polymorphism	Genotype			
	Heterozygote		Homozygote	
	Case (%)	Controls (%)	Case (%)	Controls (%)
rs3740400	74.36	71.88	25.64	28.13
rs10748835	67.50	67.65	32.50	32.35
rs3740393	84.21	76.92	15.79	23.08
rs11191439	93.33	87.50	6.67	12.50
rs429358	92.86	87.50	7.14	12.50
rs429358	24	12	4	4

**Table 3:** Haplotypes identified for the *AS3MT* SNPs tested in pregnancy loss cases and controls.

Haplotype	Alleles *	No. of Cases (%)	No. of Controls (%)
H001	G, C, G, C, A, G, T	6 (12)	12 (24)
H002	A, C, T, T, G, G, T	7 (14)	13 (26)
H003	G, T, G, C, A, C, T	7 (14)	6 (12)
H004	G, C, G, T, A, G, C	6 (12)	4 (8)
H005	G, C, G, C, A, C, T	2 (4)	2 (4)
H006	G, C, G, C, A, G, C	2 (4)	2 (4)
H007	A, C, T, C, A, C, T	1 (2)	2 (4)
H008	G, C, G, C, A, C, T	1 (2)	2 (4)
H009	A, C, G, C, A, G, T	2 (4)	0 (0)
H010	G, C, G, C, A, C, T	2 (4)	0 (0)
H011	G, T, G, C, A, C, C	2 (4)	0 (0)
H012	A, C, G, C, A, C, T	1 (2)	0 (0)
H013	A, C, G, C, G, G, C	1 (2)	0 (0)
H014	A, C, T, T, A, G, T	0 (0)	1 (2)
H015	A, C, T, T, G, G, T	1 (2)	0 (0)
H016	G, C, G, T, A, C, T	1 (2)	0 (0)
H017	G, C, G, C, A, C, C	1 (2)	0 (0)
H018	G, C, G, C, A, G, C	0 (0)	1 (2)
H019	G, C, G, C, A, G, T	1 (2)	0 (0)
H020	G, C, G, T, G, G, T	1 (2)	0 (0)
H021	G, C, G, T, G, G, T	0 (0)	1 (2)
H022	G, C, T, C, A, G, C	1 (2)	0 (0)
H023	G, C, T, T, A, G, T	0 (0)	1 (2)
H024	G, C, T, T, G, G, T	0 (0)	1 (2)
H025	G, C, G, T, A, G, C	1 (2)	0 (0)
H026	G, C, G, G, A, C, C	1 (2)	0 (0)
H027	G, C, G, T, A, G, T	1 (2)	0 (0)
H028	G, C, G, T, A, G, C	0 (0)	1 (2)
H029	G, C, G, T, A, G, T	1 (2)	0 (0)
H030	G, T, G, C, A, C, T	0 (0)	1 (2)

* the single-nucleotide polymorphisms (SNPs) are in the following order from left to right: rs7085104, rs3740390, rs3740400, rs1046778, rs10748835, rs3740393, rs11191439.

## Data Availability

The data presented in this study are available on request from the corresponding author.
